# Gabapentin may promote language development in a pediatric patient with autism spectrum disorder: a case report

**DOI:** 10.3389/frcha.2026.1685978

**Published:** 2026-03-05

**Authors:** Tobias Kremsmayer, Robert Blakey, Hugo Hidrogo, Nikolas Mata-Machado

**Affiliations:** 1 University of Illinois Chicago College of Medicine, Chicago, IL, United States; 2 Psychology, University of Bath, Bath, United Kingdom; 3 Pediatric Neurology, University of Illinois Chicago, Chicago, IL, United States

**Keywords:** autism spectrum disorder, cognitive defusion, gabapentin, language development, neuropathic pain

## Abstract

At this time, there is no report of how gabapentin may promote language development in children with autism spectrum disorder (ASD) experiencing neuropathic pain. A clinically significant increase in expressed vocabulary, around 10 words when gabapentin was prescribed to around 150 words at the 6-month follow-up, was observed in a child with ASD. This was likely due to improved symptoms of neuropathic pain, which could have allowed the patient to more effectively focus on language acquisition. Given that speech therapy had failed for years at that time to improve the patient's vocabulary and had been discontinued prior to and during the observed increase in expressive vocabulary, it was hypothesized a more direct neural effects of gabapentin could have contributed to the increase in verbal fluency. For instance, one could hypothesize that an increase in tonic, inhibitory conductance in neurons and increased stabilization of the neuronal membrane potential could negate atypical oscillatory activity observed in patients with ASD, thereby allowing for more effective learning processing. Neurodevelopmental outcomes following this reduction of atypical oscillatory activity may be mediated by thought differentiation, or cognitive defusion. Rather than cognitive defusion being an instructed state of mind as it is in psychotherapy research, it may be increased resolution to perception that is interoceptive awareness with large language models (LLMs) in neurodevelopment of the psychophysics of the neural effect, with clinical implications for the treatment of ASD.

## Introduction

Autism spectrum disorder (ASD) is characterized by persistent deficits in social interaction (e.g., delays in language development), as well as restricted and repetitive patterns of behaviors (e.g., motor stereotypies). These traits must manifest in the early developmental period and cause clinically significant impairment (1).

As it pertains to this case, the pathophysiology of language deficits in ASD is multifaceted. First and foremost, structural brain abnormalities, specifically, changes in grey matter volumes, cortical thickness and gyrification in language-related areas are thought to cause language development delays in ASD (2). Furthermore, patients with ASD exhibit reduced connectivity in language networks, particularly between inferior frontal and superior temporal regions (3). Atypical neural oscillatory activity (4), dysfunction in the auditory brainstem (5), and abnormal cortical processing of speech sounds are also thought to contribute (6).

Comorbid medical conditions, specifically MTHFR polymorphism, may also cause delays in language development. MTHFR converts 5, 10-methylenetetrahydrofolate to 5-methyltetrahydrofolate, which in turn is essential for the re-methylation of homocysteine to methionine (7). Mutations in the MTHFR gene can decrease enzyme activity, which may result in elevated homocysteine levels. Hyperhomocysteinemia is associated with increased oxidative stress and endothelial dysfunction, which can contribute to the development of neuropathic pain. Increased homocysteine concentrations can also result in the formation of peroxynitrite radicals, which can damage neurons (8–10). Furthermore, hyperhomocysteinemia can impair methylation processes, affecting myelin synthesis and further exacerbating neuropathic symptoms (11).

Gabapentin's mechanism of action is not fully understood as the medication is structurally related to the neurotransmitter gamma-aminobutyric acid (GABA), but has been shown not to affect GABA binding, uptake or degradation (12). Instead, gabapentin exhibits high-affinity binding to the α2δ subunit of voltage-gated calcium channels, thereby inhibiting calcium channel transfer to the cell membrane, which in turn decreases calcium influx into neurons and the release of excitatory neurotransmitters (e.g., glutamate, norepinephrine, and substance P) implicated in neuropathic pain. In binding the α2δ subunit of voltage-gated calcium channels, gabapentin also decreases the synaptic targeting and activity of N-methyl-D-aspartate (NMDA) receptors, which further increases the medication's analgesic effect in the treatment of neuropathic pain (13). Other mechanisms of action of gabapentin include stabilization of the neuronal membrane potential and increased tonic, inhibitory conductance in neurons (14, 15). As a result, gabapentin has been shown to significantly reduce the severity of neuropathic pain in children (16–18).

## Case presentation

The patient is a 13-year-old male with ASD, MTHFR polymorphism, trisomy 20, gastroparesis and dysautonomia who has been observed by neurology since he was less than two years old. The patient's birth was unremarkable without complications. However, the patient's mother has a history of epilepsy for which she continued to take phenytoin throughout the pregnancy. Despite this, she experienced breakthrough seizures for the last month of her pregnancy. Additionally, despite regular enoxaparin injections for a history of thrombosis, the patient's mother experienced four thrombotic events during the pregnancy. The patient's family also experienced significant personal turmoil in the years leading up to the pregnancy with unemployment of both parents in the first two years of the patient's life. There is family history of hearing loss (mother), spina bifida (mother, sister), speech and language delays (sister), and Tay-Sachs disease (brother). There is no family history of attention deficit hyperactivity disorder (ADHD), Down syndrome, autism, or cerebral palsy.

At the age of 19 months, the patient was evaluated by speech therapy due to concern of delay. The Rossetti Infant-Toddler Language Scale was administered, which showed severe impairment in receptive and expressive language skills compared with age-matched peers. More specifically, the patient exhibited skills at the 6–9-month-old level in the criterium of interaction-attachment and at the 3–6-month-old level with emerging 6–9-month-old skills in pragmatics, play, comprehension, and expression. At this time, the patient's spoken vocabulary consisted of “mamma” and “dadda” although not used purposefully. Based on this assessment, he was diagnosed with receptive/expressive language disorder with a decreased ability to communicate his wants and needs. 60 min of speech therapy a week was recommended at that time, which would continue until the age of 4 years and 5 months.

At the age of 22 months, the patient was able to verbally express 3 words per pediatric neurology. At the age of 2 years and 1 month, the patient's pediatrician administered the Early Intervention Medical Diagnostic Evaluation, which showed he follows simple, single-step instructions without gesture (receptive language), exhibits difficulties initiating and sustaining conversation (semantics & pragmatics), overstuffing with gagging and placing non-food items in mouth (oral motor) as well as decreased eye contact with unfamiliar people and avoidance of groups of children (social relatedness/reciprocity). Problematic behaviors included PICA and mouthing, while sensory abnormalities were reported as hypersensitivities to certain pitches. The patient also exhibited repetitive motor behaviors, including spinning, hand-flapping, and hand-finger movements along with obsessive/compulsive paint licking at home. Expressed vocabulary at this time consisted of around 6 words. Screening Test for Autism in Toddlers (STAT) was attempted but discontinued due to insufficient participation. Based on this assessment, the patient was diagnosed with global developmental delay and feeding disorder.

Two months later, a 24 h EEG administered at the age of 2 years and 3 months per pediatric neurology was abnormal with generalized burst of spikes and wave activity. The patient's mother denied any seizure activity. An MRI of the brain at the same time was unremarkable. It was noted that the patient had not progressed in his speech despite continued therapy.

The patient was re-evaluated by speech therapy at the age of 3 years and 4 months. The assessment re-emphasized receptive/expressive language disorder characterized by decreased comprehension of age-appropriate morphosyntax, limited verbal output with an expressive vocabulary of 4 words, and functional limitations mainly consisting of a decreased ability to communicate with family and peers in his daily environment. Eight months later, at the age of 4 years, the patient's pediatric neurologist noted failure to advance in speech with an expressive vocabulary of 3 words.

At the age of 4 years and 5 months, the patient underwent a multidisciplinary evaluation, including the Autism Diagnostic Observation Schedule (ADOS) and Beery-Buktenica Developmental Test of Visual-Motor Integration (Beery VMI) administered by developmental pediatrics. On the ADOS, the patient scored 6 (communication), 8 (reciprocal social interaction), 4 (play), and 3 (stereotyped behaviors and restricted interests). He scored 75 on the Beery VMI. It was noted the patient exhibited deficits in social-emotional reciprocity, nonverbal communicative behaviors, and in developing, maintaining and understanding relationships. Additionally, he exhibited at least two types of repetitive patterns of behavior including stereotyped motor movements, insistence of sameness including intolerable adherence to routines, and hyperreactivity to sensory input. Overall, this assessment was consistent with the diagnosis of autism spectrum disorder. Within the same week, the patient was re-evaluated by speech therapy who reported a consistent, expressive vocabulary of around 10 words. Over the next month, the patient underwent audiological examination, including Stacked Auditory Brainstem Response (SABR), and ophthalmologic examination without any abnormalities noted.

At the age of 4 years and 11 months, the patient and his mother returned for treatment of constant pain in the bilateral feet, which was diagnosed as neuropathic pain. Gabapentin was prescribed with an initial dose of 64 mg (2.5 mg/kg × weight of 25.6 kg) once at nighttime. Over the following six months, the medication was up titrated to 125 mg twice daily to reach therapeutic dosing without significant side effects while the patient's weight remained stable. During this time frame, speech therapy was discontinued due to lack of progress. Shortly after starting the medication, during a 3-month follow-up visit, the mother stated the patient “has been talking more since he started gabapentin”. When the patient returned for a follow-up at the age of 5 years and 5 months, his expressive vocabulary had increased to around 150 words with the provider noting “much improved speech since starting Neurontin”, “not receiving speech therapy currently”, and “discharged from speech therapy prior to speech increase”. During this encounter, the patient's mother stated, “the medication prevents him from focusing on the pain and allows him to more fully engage in communication”. Over the next year, gabapentin was up titrated to 300 mg twice daily due to continued pain with a stable patient weight of around 25 kg and without additional progress in language. During a follow-up visit with pediatric neurology at the age of 6 years and 5 months, the expressive vocabulary was noted to remain around 150 words.

The patient is currently in 7th grade with stable language and communication (expressive vocabulary of around 150 words), taking 300 mg of gabapentin three times daily without significant side effects. The patient's clinical course is summarized in Table 1. Of note, the patient was reported to have experienced sleep difficulties as early as 2 years of age with an average of 4–6 h of a night. This was attributed to dysautonomia due to concurrent symptoms of episodic flushing and overheating. This pattern remained stable throughout the above-described time course despite treatment with melatonin and clonidine.

**Table 1:** Clinical course.

Patient age	Event/expressive vocabulary
19 months	Expressive/receptive language disorder diagnosis, 2 words
22 months	3 words
2 years, 1 month	Global developmental delay & feeding disorder diagnosis, 6 words
2 years, 3 months	Abnormal 24 h EEG, normal MRI
3 years, 4 months	Re-evaluation by speech therapy reemphasizing receptive/expressive language disorder, 4 words
4 years	3 words
4 years, 5 months	Diagnosis of ASD, 10 words
4 years, 6 months	Audiological and ophthalmological evaluation without abnormalities
4 years, 11 months	Gabapentin (64 mg QD) started for neuropathic pain
5 years, 5 months	Gabapentin (125 mg BID), 150 words
6 years, 5 months	Gabapentin (300 mg BID), 150 words
13 years	Gabapentin (300 mg TID), 150 words

## Discussion

To date, there are no reports on pediatric patients with ASD who experience neuropathic pain benefiting from gabapentin treatment nor how this medication may promote language development in this population. In the case of this patient, it is likely that gabapentin allowed for an increase in expressive vocabulary by reducing the symptoms of neuropathic pain (17, 18). This relief in symptom may have allowed the patient to focus more effectively on language acquisition. The improvement in spoken language was significant with around ten words uttered at the time gabapentin was prescribed and having increased to around 150 words at the six-month follow-up visit. Speech therapy had been a consistent factor that had failed to improve the patient's speech. The patient's speech therapy had also been discontinued due to a lack of progress prior and during the observed increase in expressive vocabulary, suggesting minimal influence on the observed improvement. The possibility of an emerging language trajectory unrelated to a medication-associated effect remains, but no evidence was found to suggest the potential for a later expressive “spurt”. Alternatively, neural effects of gabapentin could have contributed to the patient's increase in verbal fluency. As described previously, a 24 h EEG administered at the age of 2 years and 3 months was abnormal, showing generalized burst of spikes and wave activity. It could therefore be hypothesized that an increase in tonic, inhibitory conductance in neurons and increased stabilization of the neuronal membrane potential mediated by gabapentin could have negated atypical oscillatory activity observed in patients with ASD, thereby allowing for more effective learning processing (4, 14, 15, 19).

Neurodevelopmental outcomes following this reduction of atypical oscillatory activity may be mediated by thought differentiation, or cognitive defusion. When thoughts expressing the negative affect of neuropathic pain are differentiated from those associated with proximal experiences such as language acquisition, greater salience can be attributed to language learning. In this case, improved neurodevelopmental outcomes appeared to reflect increased focus on language acquisition through reduced affective interference. Cognitive defusion increases conceptual distance between language and negative affective attributions, enabling perspectives on language with diminished negative valence. Learning and affective valence are therefore central to language acquisition: attributing mentalized affect to language reflects neural learning processes within self-referential mental states, or mentalization. This supports the restoration of social cognition, counteracting sociocognitive decline associated with social atrophy. Mentalizing capacity may be measurable by large language models (LLMs) through domain-general prediction of neurodevelopmental stages. The extent to which gabapentin enhances thought differentiation, thereby improving attribution of perspective to language, may inform LLM-based conceptualizations of neurodevelopmental outcomes. Further research, including regular quantitative pain scores and neurophysiological measures, such as EEG/MEG, is needed to determine whether gabapentin may influence self-referential processing mediating increased attention to proximal experience (e.g., language acquisition in ASD) and contribute to improved neurodevelopment in social atrophy (19).

## Conclusion

Currently, there are no studies or case reports describing how gabapentin could promote language development in children with ASD. Based on this case, the medication likely allowed for improved language acquisition due to relief of neuropathic pain. However, the possibility of an emerging language trajectory unrelated to a medication-associated effect remains. Alternatively, neural effects of gabapentin could have contributed to the patient's increase in verbal fluency, but more research is required to elucidate whether an emergent neural effect may have taken effect: rather than cognitive defusion being an instructed state of mind as it is in psychotherapy research, it may be increased resolution to perception that is interoceptive awareness with LLMs in neurodevelopment of the psychophysics of the neural effect, with clinical implications for the treatment of ASD.

## Data Availability

The raw data supporting the conclusions of this article will be made available by the authors, without undue reservation.
